# Effectiveness of a Community Health Worker-Led Intervention on Knowledge, Perception, and Prostate Cancer Screening among Men in Rural Kenya

**DOI:** 10.1155/2022/4621446

**Published:** 2022-08-08

**Authors:** Ruth Gathoni Mbugua, Simon Karanja, Sherry Oluchina

**Affiliations:** ^1^Mount Kenya University, Department of Community Health, P.O. Box 342-01000, Thika, Kenya; ^2^Jomo Kenyatta University of Agriculture & Technology, School of Public Health, P.O Box 62 000-00200, Nairobi, Kenya; ^3^School of Nursing, Jomo Kenyatta University of Agriculture & Technology, P.O Box 62 000-00200, Nairobi, Kenya

## Abstract

**Background:**

Globally, an increase in mortality from prostate cancer (PC) remains a big challenge with disparities existing with a slight preponderance among men in low and middle-income countries. Prostate cancer is a leading cause of mortality among men in sub-Saharan Africa. In Kenya, despite the majority of men presenting with advanced prostate cancer for treatment, knowledge and screening for prostate cancer is low. The study aimed to examine the effectiveness of a community health worker-led education intervention on knowledge, perception, and PC screening.

**Methods:**

This was a quasiexperimental study among Kenyan men aged 40–69 years. The intervention site was Gatundu North subcounty and the control site was Kiambu subcounty in Kiambu County. Stratified random sampling was applied to select 288 respondents per arm of the study. We used a pretested interviewer-administered questionnaire to collect data at baseline and 6 months postintervention. Pearson's chi-square test was used for data analysis.

**Results:**

Awareness of prostate cancer significantly increased postintervention (*P* < 0.05). The proportion of respondents who had good knowledge of prostate cancer increased significantly from 49% to 76.4%(*P* < 0.05) in the intervention arm. The proportion of respondents with a high perception of self-vulnerability increased significantly from 26% to 42.1% (*P* < 0.05). The proportion of men who had undergone PC screening significantly increased from 4.5% to 20.4% (*P* < 0.05) in the intervention arm. In postintervention, there was a statistically significant difference in the proportion of men screened for prostate cancer in the intervention and control arm (*P* < 0.05).

**Conclusion:**

Health education by community health workers during household visits increased awareness and knowledge, perception, and uptake of PC screening. Utilization of community health worker delivered education is an effective strategy that requires to be adopted to enhance screening.

## 1. Introduction

Globally, the increase in mortality from prostate cancer (PC) remains a big challenge with disparities existing with a slight preponderance among men in low and middle-income countries. Prostate cancer is the leading cancer among males in sub-Saharan Africa (SSA) and is estimated to have contributed to 40,051 deaths and 77,295 new cases in 2020. The higher mortality rates reported in SSA in comparison to other continents are estimated to double by the year 2040 [1]. The disparity in mortality may be attributed to rising incidence, limited access to screening and treatment, and socioeconomic and cultural dynamics in existence [2]. The age-adjusted death rate is 24.17 per 100,000 of the population while the estimated average survival rate of men in SSA is 55.3%. A majority of the patients present in advanced stages has contributed to decreased survival rates [3]. The Global Burden of Disease study 2017 estimated that disability-adjusted life years from PC increased by 127.2% from 1990 to 2017 in SSA [4]. The burden of PC cancer in Kenya just like other African countries is on the rise as it was ranked as the most common cancer among males at a 5-year (all ages) prevalence rate of 21.83 per 100,000 and an age-standardized incidence rate of 39.9 per 100,000 in 2020 [1, 5, 6].

The reduction in disparities regarding mortality of men with PC is highly dependent on early diagnosis [1, 7]. Prostate cancer screening remains a much-debated issue globally, nevertheless, one agreement has been the utilization of shared decision-making among well-informed at-risk men [8]. The available statistics show low PC screening rates among men in SSA despite them having a higher risk of developing PC [2,9]. This has been attributed to low knowledge and negative beliefs [10, 11]. The Ministry of Health, Kenya, rolled out cancer screening guidelines that recommend shared decision-making during PC screening among men aged 40 to 69 years [5]. However, the rate of PC screening remains low with an estimated average of 793 men screened per year. The majority of men screened are between ages 55 and 65 years [9]. A population-based survey in Kenya indicated that only 4.3% of men aged 40–44 years and 2.6% of men aged 45–49 years were screened for PC. Men from the rural areas reported lower levels of PC awareness and screening [12]. Majority of the men present for treatment with advanced disease, which contributes to the rise in mortality which has been attributed to the low levels of screening [5]. The cancer diagnostic services are available at the secondary (county) and tertiary (national) referral hospitals. Cancer treatment facilities are inadequate and mainly found in the urban centers [13]. The most commonly used methods for PC screening include prostate-specific antigen (PSA) test and digital rectal examination [9].

Globally, the shortage of healthcare workers remains a major impediment to the achievement of Universal Health Coverage (UHC). This may worsen the aforementioned disparities in mortality from PC emanating from health inequalities in the affected populations. This calls for the utilization of alternative strategies to address the shortage of healthcare workers to increase awareness and knowledge of men to overcome barriers to screening, especially in low and middle-income countries. Task shifting in low-resource settings is a feasible strategy in the reduction of cancer burden, especially in African countries [14]. Community health workers (CHWs) may be the magic bullet to the existing healthcare workers shortage. Community health workers are uniquely positioned to provide healthcare services in the communities they live. These community resource persons can improve access to culturally appropriate healthcare among underserved communities [15].

Kenya is a signatory to Astana Declaration (2018) and has adopted primary healthcare as the approach to deliver UHC. The first level of health service delivery in the health system in Kenya is the community health service. Community health is implemented through a community health unit (CHU) that serves approximately 5,000 people and is constituted of 10 community health workers (CHWs) and 1 community health assistant (CHA). The CHWs provide preventive, curative, promotive, and rehabilitative services in the community [16]. Community health worker interventions have been rendered as cost-effective strategies to enhance cancer screening behaviors, especially in underserved populations [17]. The CHWs can be utilized to increase awareness of prostate cancer to circumvent the already existing shortage of healthcare workers.

In Kenya, despite the increase in mortality from PC, several studies conducted show low levels of knowledge and PC screening rates [18,19]. Given the increased presentation of PC patients in advanced stages in Kenya, and the paucity of community-based interventions to address this public health puzzle. The purpose of the study was to examine the effectiveness of a community health worker-led education intervention on knowledge, perception, and PC screening among at-risk men in a rural community in Kenya.

## 2. Materials and Methods

**Figure 1:**
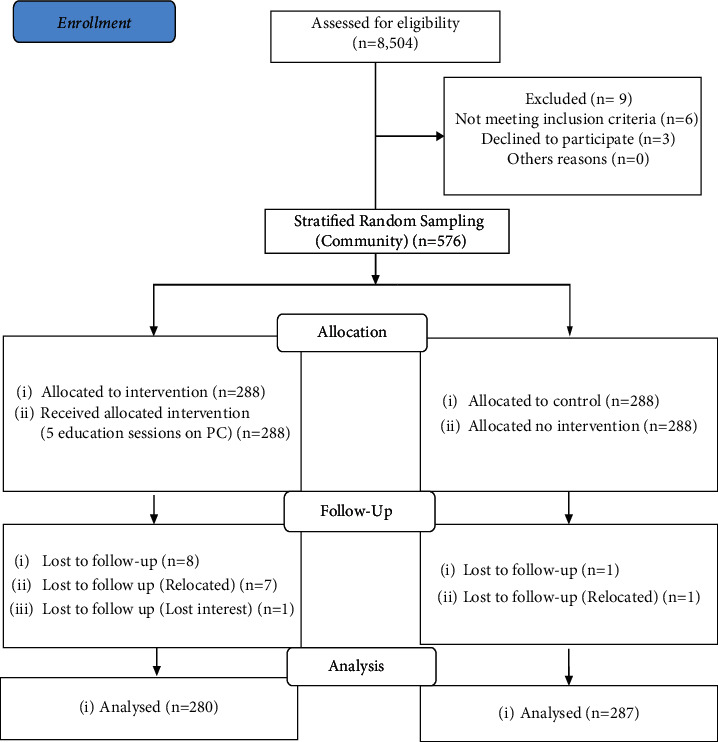
CONSORT diagram of study participants in the arms of the study.

The study design was quasiexperimental (pretest and posttest). The study was conducted from April to October 2019 in Kiambu County within the Central region of Kenya. To avoid contamination of the study, two different subcounties were selected within the study area. Gatundu North subcounty was the intervention arm and the Kiambu subcounty was the control arm. Additionally, to mitigate bias that may be introduced by the utilization of the study design, the study subjects were randomly selected and assigned to the intervention and control arms. The target population was men aged 40–69 years who were considered eligible for PC screening in line with the Ministry of Health screening guidelines [5]. Stratified random sampling was applied to select participants. The strata was a community health unit (CHU), which is a geographically defined unit in the community that serves a population of approximately 5,000 people. All the CHUs in the study site were included in the study. Men aged 40–69 years were listed per CHU in the study area. A table of random numbers was used to select 288 men who met the inclusion criteria per arm of the study. The sample size was determined using the formula for comparing proportions [20]. The exclusion criteria were men with a confirmed diagnosis of PC or who had major medical illnesses that would preclude them from receiving PC screening. Simple random sampling was used to select 3 CHWs per CHU. A list of all the trained CHWs in the 11 CHUs in the intervention arm was generated. All the inactive or dropped-out CHWs were excluded from the study. A total of 33 CHWs were selected from the 11 CHUs in the intervention site to deliver health education in the selected households. The intervention arm participants received a CHW face-to-face health education in their households (Figure 1).

A pretest was done in Thika subcounty among 29 men which represented 10% of the intervention study population. The Cronbach's alpha results for items used to measure knowledge (0.73) and perception of self-vulnerability (0.71) were reliable in measuring their respective variables (Table 1). The research assistants were identified and underwent intensive training on the use of the questionnaire before the research to reduce interview bias and posttests were done in the two arms of the study to assess for differences in outcomes following the health education intervention. Pre-intervention, a baseline assessment of awareness and knowledge, perception of self-vulnerability, and uptake of PC screening in the intervention and control arm was conducted.

### 2.1. Intervention

A training guideline for CHWs based on the Ministry of Health, Kenya Community Health Workers training Module 13, was developed by the principal investigator. The training guideline was validated by a panel of experts in the Ministry of Health to ascertain its appropriateness and authorization sought. The CHWs were trained on prostate cancer for two (2) days. To ensure interventional fidelity, the training of CHWs included role play and the use of a standard training tool kit by CHWs during health education. The trained CHWs conducted home visits and delivered health education to the selected participants in the intervention arm of the study. The health education included: definition and classification, symptoms, risk factors, diagnosis, management, and prevention of prostate cancer. A total of 33 CHWs were recruited and each delivered health education in 8-9 households. An initial session of health education was delivered to the enrolled participants in their households and monthly follow-up sessions for six months. A household visit checklist was developed which was used as a monitoring tool for the initial and follow-up household visits.

**Figure 2:**
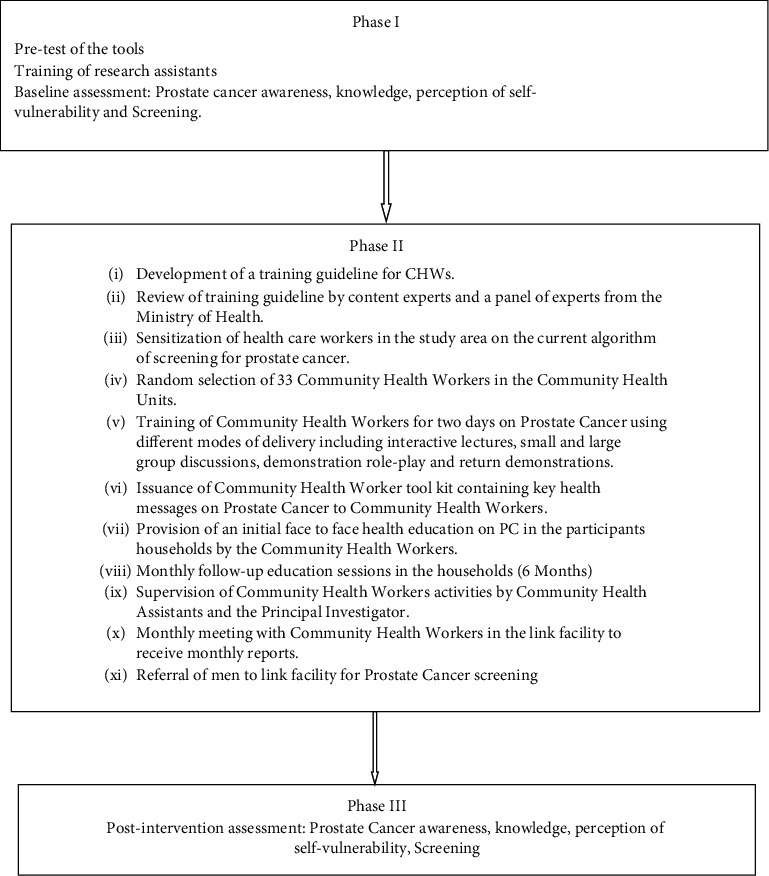
Diagrammatic representation of the community-based health education intervention.

Upon completion of the visit, the checklist was signed by the CHW, the participant received by the CHA of the particular community health unit (CHU) and forwarded to the subcounty public health officer. Supervision of the activities in the households was done by the principal investigator and the CHAs of the particular CHUs. Monthly meetings were held with the CHWs at Igegania Hospital. The aim of the meetings entailed giving a detailed report on the activities of the month per household and the outcomes. A small reimbursement fee to cater for the transport of 10 dollars was given to CHWs during each monthly meeting. A posttest was carried out after 6 months using the same tool. The control group did not receive any intervention (Figure 2).

### 2.2. Data Collection

A pretested interviewer-administered questionnaire was used to collect data at baseline and six months after the intervention in the study arms. The questionnaire assessed the sociodemographic characteristics, knowledge, awareness, perceived self-vulnerability, and history of PC screening among the respondents.

Knowledge was measured using 13 items modified from the Integrative Model of Prostate Cancer Disparities, a validated survey instrument [21]. The construct of Perception of self-vulnerability was assessed using 11 items that were assessed for absolute vulnerability, conditional vulnerability, and cancer worry [22] (Gerrard et al.) based on a five-point Likert scale (Table 1).

At postintervention, which was carried out 6 months after the education intervention, data were collected using the same questionnaire to assess for differences in outcomes (knowledge and awareness, perception of self-vulnerability, and screening) in both arms of the study.

### 2.3. Data Analysis

Data were analyzed using Statistical Package of Social Sciences (SPSS) version 22. The proportion of men with a history of PC screening was assessed. Knowledge was measured using 13 items [11] and perception of self-vulnerability was assessed using 11 items using a Likert scale ranging from strongly disagree (1) to strongly agree (5). The scores were computed for the level of agreement at baseline and postintervention. The proportions of the variables were compared at baseline and postintervention using Pearson's chi-square. A *P*-value of <0.05 was considered significant.

### 2.4. Ethical Consideration

Ethical clearance was sought from the Jomo Kenyatta University of Agriculture and Technology Institutional Ethics Review Committee (JKU/2/4/896B). Authorization to conduct the study was also sought from the Kenya Ministry of Health. Participants' confidentiality and autonomy were ensured. The benefits and purpose of the study were explained and informed consent was sought from the participants. The participants were not coerced to participate in the study or take up screening. The privacy of men was considered during the health education sessions by the CHWs in the households.

## 3. Results

### 3.1. Sociodemographic Characteristics

The sociodemographic characteristics of the intervention and control arm respondents are given in Table 2. At baseline, 288 men aged 40–69 years were recruited per arm of the study. At postintervention, the response rate in the intervention arm was 97.2% (280), while in the control arm it was 99.7% (287).

### 3.2. Prostate Cancer Awareness, Knowledge, and Perception of Self-Vulnerability

In postintervention, the proportion of respondents who had heard about PC increased significantly from 83.3% to 99.3% (*P* < 0.05) in the intervention arm in comparison to the control arm where there was a slight change from 83.7% to 83.3% (*P* < 0.05). There was a significant difference in the intervention and control arms of the study at postintervention (*P* < 0.05). In postintervention, awareness on PC screening in the control arm was 29.4% compared to 90% in the intervention arm (*P* < 0.05) (Table 3).

The proportion of respondents who had good knowledge of prostate cancer increased significantly from 49% to 76.4% (*P* < 0.05) in the intervention arm, whereas in the control arm it increased slightly from 57.3% to 62.7% (*P*=0.202). The proportion of respondents with a high perception of self-vulnerability increased significantly from 26% to 42.1% (*P* < 0.05) in the intervention arm whereas in the control arm it increased from 23.6% to 24.0% (*P*=0.734) (Table 4).

### 3.3. Prostate Cancer Screening

Table 4 provides that postintervention, the proportion of men who had undergone PC screening significantly, increased from 4.5% to 20.4% (*P* < 0.05) in the intervention arm, whereas in the control arm it slightly increased from 5.6% to 6.3% (*P*=0.716). Study findings indicate that there was a statistically significant difference in the proportion of men screened for prostate cancer in the intervention arm and control arm postintervention (*P*=<0.05).

## 4. Discussion

The health education intervention assessed in our study was the utilization of community health workers to deliver health education on PC. This involved the conducting of home visits by CHWs and delivery of information on PC to at-risk men to increase their knowledge on PC and enhance PC screening decision-making. The selection of the strategy to be utilized is paramount to ensure the success of an education intervention. Strategies utilized especially among African men require to be culturally tailored and should enhance ownership [23]. Health education delivered by CHWs proved to be an effective strategy as it increased knowledge and awareness, self-vulnerability, and PC screening. Community health workers are selected from the communities they serve and hence may be well positioned to overcome the cultural barriers that exist in the community [14].

The level of awareness and knowledge on PC improved significantly in the intervention arm at postintervention. A study conducted among medically underserved retired men in Iran who utilized health education reported a significant increase in knowledge in the intervention arm. Another study conducted among Jamaican men found that there was an improvement in knowledge of PC risk factors and screening among men following an education intervention [24]. The effectiveness of community-based interventions on knowledge on PC has been reported across countries [17, 25, 26]. A similar study reported a significant increase in prostate cancer knowledge following a community health worker-led intervention among black men [27]. This indicates that culturally relevant health education delivered by the CHWs can enhance knowledge on PC. The improvement of knowledge among men has been anticipated to enhance the decision-making process regarding PC screening [28].

The study findings showed a significant increase in the perception of self-vulnerability among the respondents in the intervention arm in comparison with the control arm. Several studies have similarly reported improvements in risk perception following an education intervention. A study conducted in Iran that assessed the influence of health belief model-based education reported a significant increase in risk perception following the education intervention [29]. Similarly, there was an increase in the perception of self-vulnerability among men in Iran following an education intervention [30]. The perception of risk is associated with prostate cancer screening [31]. The more men are aware regarding prostate cancer, the higher the likelihood to perceive themselves at risk and hence take up screening. The perception of unrealistic optimism, where one has a false belief that they are less vulnerable to a condition in comparison to other people, is a significant deterrent to the uptake of cancer screening.

The uptake of PC screening significantly increased following face-to-face household visits by CHWs in the study. These findings are not unique to our current study, other studies utilizing different forms of education interventions have reported a similar increase in the uptake of PC screening. For instance, a study conducted in the Shiraz community in Iran reported an increase in PC screening in the intervention group after an education intervention [32]. The findings of the study are congruent with a study conducted among black men which found that a community health worker-led education intervention significantly enhanced screening [25, 33]. Similarly, studies conducted in Kenya and Nigeria found that cervical cancer screening increased significantly following the utilization of a community-based education intervention [34, 35].

Community health workers have been recognized globally as culturally competent ‘health brokers' [36]. The utilization of CHWs for the prevention of PC may go a long way in low-resource countries like Kenya as community-based health worker interventions have been rendered as cost-effective strategies for the improvement of cancer screening behaviors especially in underserved populations [17, 37]. The uptake of PC screening is a complex medical decision that requires knowledgeable men to enhance informed decision-making [38, 39]. It is therefore imperative for the consideration of the provision of culturally acceptable education to men to enhance their decision-making. The utilization of CHWs to enhance uptake of PC screening is a culturally relevant strategy that can be explored as CHWs are already familiar with the community and would additionally aid in circumventing the shortage of healthcare workers, especially in developing countries. The findings of this study suggest that health education delivered by CHWs during household visits may be vital in shaping the knowledge and uptake of PC screening in Kenya. The improvements observed may be enhanced over time if CHWs continue educating men about PC during their routine household visits in the community. The long-term goal of health education shall be early detection and decrease in mortality related to PC among at-risk men in the community. The scale-up of this community-based intervention is feasible through leveraging on the existing primary healthcare structures in Kenya.

This study is not without limitations. First, it was conducted in a rural community in the central region of Kenya so the results may be generalized to other populations with caution. Second, the study utilized a quasiexperimental design hence randomization which is the gold standard in experimental studies was not done which may have introduced bias. Nonetheless, the sample size selected was large and subjects were randomly sampled from two different subcounties for the intervention and control group to minimize bias.

## 5. Conclusion

Health education by community health workers during household visits increased awareness and knowledge, perception, and uptake of PC screening. Community-based health education interventions can be utilized to increase awareness and knowledge and screening. The scaling up of community health worker-led education interventions is a feasible cost-effective strategy that requires to be considered for the prevention and control of prostate cancer.

## Figures and Tables

**Table 1:** Questions for assessment of knowledge and perception of self-vulnerability.

*Knowledge on prostate cancer*	
(a)	I will be able to know I have prostate cancer immediately through the symptoms I experience.
(b)	Younger men are more likely to get prostate cancer than older men
(c)	Having somebody in your family with prostate cancer increases the chance of one getting prostate cancer
(d)	Eating red meat increases the risk of a men developing prostate cancer
(e)	Eating vegetables increases the risk of a men developing prostate cancer
(f)	A man with many sexual partners is more likely to develop prostate cancer
(g)	A man can prevent themselves from getting prostate cancer by not smoking cigarettes/using tobacco.
(h)	Prostate cancer disease is curable
(i)	Prostate cancer can cause death if it is left untreated
(j)	Early testing for prostate cancer cannot tell if one has prostate cancer
(k)	Prostate cancer diagnosed early through testing increases survival
(l)	All adult men should undergo prostate cancer screening
(m)	Men should undergo prostate cancer screening once in their lifetime

*Perception of self-vulnerability*	
(a)	In my opinion prostate cancer is not a common disease
(b)	At my age, I do not need to get screened for prostate cancer
(c)	I believe that I am at risk of getting prostate cancer.
(d)	I believe that I am at a higher risk of getting prostate cancer than other men
(e)	Compared to other diseases, prostate cancer screening is not important to me
(f)	It is likely that I will get prostate cancer in future
(g)	I am worried about having prostate cancer
(h)	I am worried about having a prostate cancer test because I do not understand what will be done
(i)	I believe having a prostate cancer test would cost too much money unnecessarily
(j)	I believe that getting a prostate cancer test would take too long at the hospital
(k)	I am too busy to undertake prostate cancer screening

**Table 2:** Sociodemographic characteristics.

Variable	Category	Control (*n* = 28), frequency (%)	Intervention (*n* = 288), frequency (%)	Total (*n* = 576), frequency (%)	Chi-square/Fishers exact
Age in years	40–49	102 (35.4)	97 (33.7)	199 (34.5)	*P*=0.502
	50–59	97 (33.7)	100 (34.7)	197 (34.2)	
	60–69	89 (30.9)	91 (31.6)	180 (31.3)	
Marital status	Married	227 (78.8)	242 (84.0)	469 (81.4)	
	Single/widowed/separated	61 (21.2)	46 (16.0)	107 (18.6)	*P*=0.468
Religion	Christian	283 (98.3)	282 (97.9)	565 (98.1)	
	Traditionalist	2 (0.7)	4 (1.4)	6 (1.0)	Exact = 0.803
	Muslim	3 (1.0)	2 (0.7)	5 (0.9)	
Education	None	4 (1.4)	2 (0.7)	6 (1)	
	Primary	89 (30.9)	91 (31.6)	180 (31.3)	*P*=0.437
	Secondary	151 (52.4)	149 (51.7)	300 (52.1)	
	Tertiary	44 (15.3)	46 (16.0)	90 (15.6)	

**Table 3:** Participants awareness on prostate cancer at baseline and postintervention.

Variable	Baseline			Postintervention		
	Intervention (*n* = 288), frequency (%)	Control (*n* = 288), frequency (%)	Chi-square	Intervention (*n* = 280), frequency (%)	Control (*n* = 287), frequency (%)	Chi-square
Ever heard of PC	240 (83.3)	241 (83.7)	*X * ^2^ = 0.013 *P*=0.911	278 (99.3)	239 (83)	*X * ^2^ = 36.607 *P* < 0.05
Ever heard of PC screening	53 (18.4)	65 (22.6)	*X * ^2^ = 0.013 *P*=0.911	252 (90)	84 (29.3)	X^2^ 58.049 *P* < 0.05

**Table 4:** Effectiveness of community-based health education on knowledge, perception of self-vulnerability, and uptake of screening.

Intervention, *N* (%)			Control, *N* (%)		
Variable	Baseline (*n* = 28), frequency (%)	Post-intervention (*n* = 280), frequency (%)	Chi-square	Baseline (*n* = 288), frequency (%)	Post-intervention (*n* = 287), frequency (%)	Chi-square
Good knowledge	141 (49%)	214 (76.4%)	*P* < 0.05	165 (57.3%)	180 (62.7%)	*P*=0.202
Poor knowledge	147 (51%)	66 (23.6%)		123 (42.7%)	107 (37.3%)	
High perception of self-vulnerability	75 (26%)	118 (42.1%)	*P* < 0.05	68 (23.6%)	69 (24%)	*P*=0.734
Low perception of self-vulnerability	213 (74%)	162 (57.9%)		220 (76.4%)	218 (76%)	
Screened	13 (4.5%)	57 (20.4%)	*P* < 0.05	16 (5.6%)	18 (6.3%)	*P*=0.716
Not screened	275 (95.5%)	223 (79.6%)		272 (94.4%)	269 (93.7%)	

## Data Availability

The data used in the study are available from the corresponding author upon request.
